# Cerebral Embolic Risk in Coronary and Structural Heart Interventions: Clinical Evidence

**DOI:** 10.1016/j.jscai.2023.100631

**Published:** 2023-03-29

**Authors:** Daniela Tirziu, Haocheng Huang, Helen Parise, Cody Pietras, Jeffrey W. Moses, Steven R. Messé, Alexandra J. Lansky

**Affiliations:** a Yale Cardiovascular Research Group, Section of Cardiovascular Medicine, Department of Internal Medicine, Yale University School of Medicine, New Haven, Connecticut; b Division of Cardiology, Department of Internal Medicine, Columbia University Medical Center, New York, New York; c St. Francis Hospital & Heart Center, Roslyn, New York; d Department of Neurology, Hospital of the University of Pennsylvania, Philadelphia, Pennsylvania

**Keywords:** cardiac procedures, cerebral embolism, coronary, DW-MRI lesions, stroke, structural heart, surgical, transcatheter

## Abstract

Surgical and endovascular procedures for coronary and structural heart interventions carry a meaningful risk of acute stroke with greatly increased likelihood of disability and long-term neurocognitive sequelae. In the last decade, transcatheter aortic valve replacement procedures have focused our attention on a spectrum of procedure-related neurologic injuries that have led to various efforts to prevent ischemic injury with the use of embolic protection devices. As the number of patients undergoing surgical and transcatheter cardiac procedures in the United States continues to increase, the risk of iatrogenic brain injury is concerning, particularly in patient populations already at increased risk of thromboembolism and cognitive decline. In this study, we reviewed the current estimates of the incidence of iatrogenic cerebral embolization and ischemic infarction after surgical and percutaneous transcatheter interventions for coronary artery disease, stenotic aortic and mitral valves, atrial fibrillation, left atrial appendage and patent foramen ovale closure. Our findings show that every year in the United States, nearly 2 million patients undergo coronary and structural heart interventions, with approximately 8000 at risk of experiencing a symptomatic stroke and 330,225 (95% CI, 249,948-430,377) at the risk of ischemic brain injury after the procedure. Given the increased use of surgical and endovascular cardiac procedures in clinical practice, the risk of iatrogenic cerebral embolism is significant and demands careful consideration through neurologic and cognitive assessments and appropriate risk mitigation.

## Highlights

• Every year, 2 million patients undergo coronary and structural heart interventions.
• 8000 patients are at risk of experiencing a symptomatic stroke after the procedure.
• Up to 430,377 patients are at risk of ischemic brain injury after the procedure.
• Ischemic brain injury is a risk factor for cognitive decline, dementia, and stroke.
• Iatrogenic cerebral embolic injury warrants longer-term neurocognitive evaluations .

## Central Illustration

**Figure:**
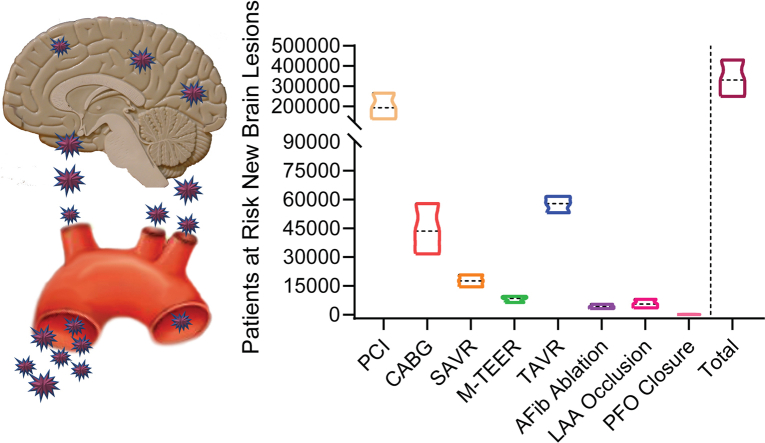

## Introduction

Each year in the United States, large numbers of patients undergo coronary and structural heart procedures. Guideline-recommended cardiac procedures are universally associated with risk of iatrogenic symptomatic (overt) stroke and subclinical (covert) neurologic injury that vary in frequency with the type of procedure. Increasing attention has been focused on the significant burden of neurologic injury detected by diffusion-weighted magnetic resonance imaging (DW-MRI) resulting from cerebral embolization as a direct consequence of cardiac instrumentation and dislodgement of atheromatous or calcific debris during intervention.^1^, ^2^, ^3^, ^4^, ^5^ Although the morbidity and mortality effect of procedural overt stroke are well documented, the long-term clinical and cognitive consequences of acutely asymptomatic covert brain injury detected by DW-MRI remain incompletely characterized.

The aim of this review was to provide current evidence of the prevalence and near-term prognosis of iatrogenic stroke and subclinical cerebral injury on DW-MRI for patients undergoing surgical and transcatheter interventions related to coronary artery disease and structural heart disease (stenotic aortic/mitral valves, atrial fibrillation [AFib], left atrial appendage [LAA], patent foramen ovale [PFO], and atrial septal defect [ASD]). The iatrogenic procedural risk of cerebral embolization may compound the inherent stroke risk of patients undergoing procedures such as LAA/PFO occlusion or AFib ablation, which emphasizes the need to better understand the incidence and prognosis of the added procedural embolic risk. Pathophysiology and proper antithrombotic management of LAA and PFO occlusion are still far from being fully understood, and these patients are at a higher risk for thrombus formation leading to peripheral arterial or cerebral embolism.^6^, ^7^, ^8^, ^9^, ^10^ The longer-term clinical implications of the ischemic burden of cardiac procedures remain poorly characterized in the absence of high-quality data.

## Data source and presentation

Coronary and structural heart interventions encompass a broad spectrum of open surgery and endovascular procedures. For the purpose of this review, data are presented for cardiac catheterization, percutaneous transluminal coronary angioplasty (PTCA), coronary artery bypass grafting (CABG), surgical aortic valve replacement (SAVR), surgical mitral valve replacement (SMVR), surgical mitral valve repair (MV repair), transcatheter mitral valve replacement, mitral transcatheter edge-to-edge repair (M-TEER), transcatheter aortic valve replacement (TAVR), AFib catheter ablation, and percutaneous closure of LAA, PFO, and ASD.

The number of each type of intervention performed annually in the United States was estimated based on the latest reports of the American Heart Association Heart Disease and Stroke Statistics,^11^ the Society of Thoracic Surgeons (STS) Adult Cardiac Surgery Database,^12^ the Society of Thoracic Surgeons—American College of Cardiology (STS/ACC) Transcatheter Valve Therapy (TVT) Registry,^13^ the National Cardiovascular Data Registry LAAO Registry,^14^ and the National Inpatient Sample (NIS),^15^^,^^16^ as indicated. The annual volume of procedures is presented by the type of intervention and overall procedure count.

The in-hospital stroke rate for each type of intervention was estimated based on published registry data including the STS Adult Cardiac Surgery Database,^12^ the STS/ACC TVT registry,^13^ the National Cardiovascular Data Registry LAAO Registry,^14^ the NIS,^15^^,^^17^ high-volume hospital data,^18^^,^^19^ and meta-analyses,^20^ as indicated. The annual number of patients with in-hospital stroke was estimated relative to the annual volume of procedures per each type of intervention and overall count.

The prevalence of procedural covert stroke for each type of intervention was estimated based on a literature search of published data in PubMed and the Cochrane Database of Systematic Reviews. The studies were selected if DW-MRI was used to identify new ischemic lesions in the early postoperative period (1-7 days) (Supplemental Appendix). Data on the occurrence of DW-MRI lesions were pooled for each type of intervention, and pooled estimates with 95% CI were calculated using the inverse-variance random effects model. The annual number of patients at risk for covert stroke detected by DW-MRI was estimated based on pooled estimates of DW-MRI lesion prevalence with 95% CI relative to the annual volume of procedures for each type of intervention and overall.

## Coronary and structural heart interventions

According to registry data, nearly 2 million patients undergo a coronary or structural heart intervention each year in the United States (Table 1). Among these, diagnostic cardiac catheterization and PTCA represent 81% of the total annual volume. Surgical procedures such as isolated CABG represent 9%, and CABG in combination with SAVR, SMVR, or MV repair are performed in 1% of patients. Nearly 2% of patients undergo surgical or transcatheter treatment of the MV. Currently, TAVR is performed in more patients than SAVR (4% vs 1%), consistent with the trend observed in the treatment of aortic stenosis in the last decade.^12^ Overall, catheter ablation for AFib and percutaneous closures of LAA, PFO, and ASD are performed in 2% of patients. Importantly, the volume of coronary and structural heart interventions performed each year is predicted to continue to grow, given the rapid utilization of emerging newer transcatheter procedures and expanding indications for TAVR from extreme– to low–surgical risk patients^13^ and M-TEER with MitraClip in patients with inoperable heart failure.^21^^,^^22^

**Table 1:** Annual volume of US patients undergoing coronary and structural heart procedures and estimated procedural symptomatic stroke.

Cardiac procedures	No. of annual US patients	In-hospital stroke (%)	Estimated annual US patients with in-hospital stroke (n)
Cardiac catheterization	1,016,000^11^	0.10^18^	1016
PTCA	480,000^11^	0.40^19^	1920
CABG	161,816^12^	1.40^12^	2265
SAVR	20,965^12^	1.20^12^	252
SAVR+CABG	14,246^12^	2.00^12^	285
SMVR	10,748^12^	1.90^12^	204
SMVR+CABG	3441^12^	3.10^12^	107
MV repair	12,570^12^	0.90^12^	113
MV repair+CABG	4153^12^	2.10^12^	87
SAVR and SMVR	2624^12^	2.10^12^	55
M-TEER	10,692^12^	0.90^20^	96
TMVR	1164^12^	1.00^20^	12
TAVR	72,991^13^	1.60^13^	1168
AFib ablation	22,000^15^	0.31^15^	68
LAA occlusion	18,000^14^	0.17^14^	3
PFO/ASD closure	1735^16^	2.9^17^^a^	50
Total	1,853,145	0.42	7729

AFib, atrial fibrillation; ASD, atrial septal defect; CABG, coronary artery bypass grafting; LAA, left atrial appendage; M-TEER, mitral transcatheter edge-to-edge repair; MV, mitral valve (surgical); PFO, patent foramen ovale; PTCA, percutaneous transluminal coronary angioplasty; SAVR, surgical aortic valve replacement; SMVR, surgical mitral valve replacement; TAVR, transcatheter aortic valve replacement; TMVR, transcatheter mitral valve replacement.

a The combined rate of stroke or transient ischemic attacks.

## Incidence of symptomatic stroke after cardiac procedures

In-hospital stroke is estimated to affect ∼8000 patients per year in the United States, representing 0.4% of the total annual procedure volume across the spectrum of patient risk (Table 1). Approximately half of these patients have a stroke after open surgical—individual or combined—procedures. Registry data showed that in 2019, the risk of stroke associated with surgical procedures such as CABG, SAVR, SMVR, and MV repair ranged from 1.2% to 3.1%,^12^ which is higher compared with percutaneous transcatheter procedures (Table 1). Another 38% of annual procedure-related strokes occur after cardiac catheterization or PTCA, which have a lower absolute risk but a higher procedural volume. TAVR contributes ∼15% of the annual procedure-related strokes. According to the data reported by the TVT registry, in 2019, in-hospital stroke rates varied from 1.6% up to 1.9% in high/extreme-risk patients.^13^ Fewer patients (<2% of total) experience stroke after structural heart procedures such as AFib catheter ablation and percutaneous closure of LAA, PFO, and ASD procedures with an estimated risk of procedural stroke of <1% (Table 1).

## Procedural covert stroke (detected by DW-MRI)

**Figure 1:**
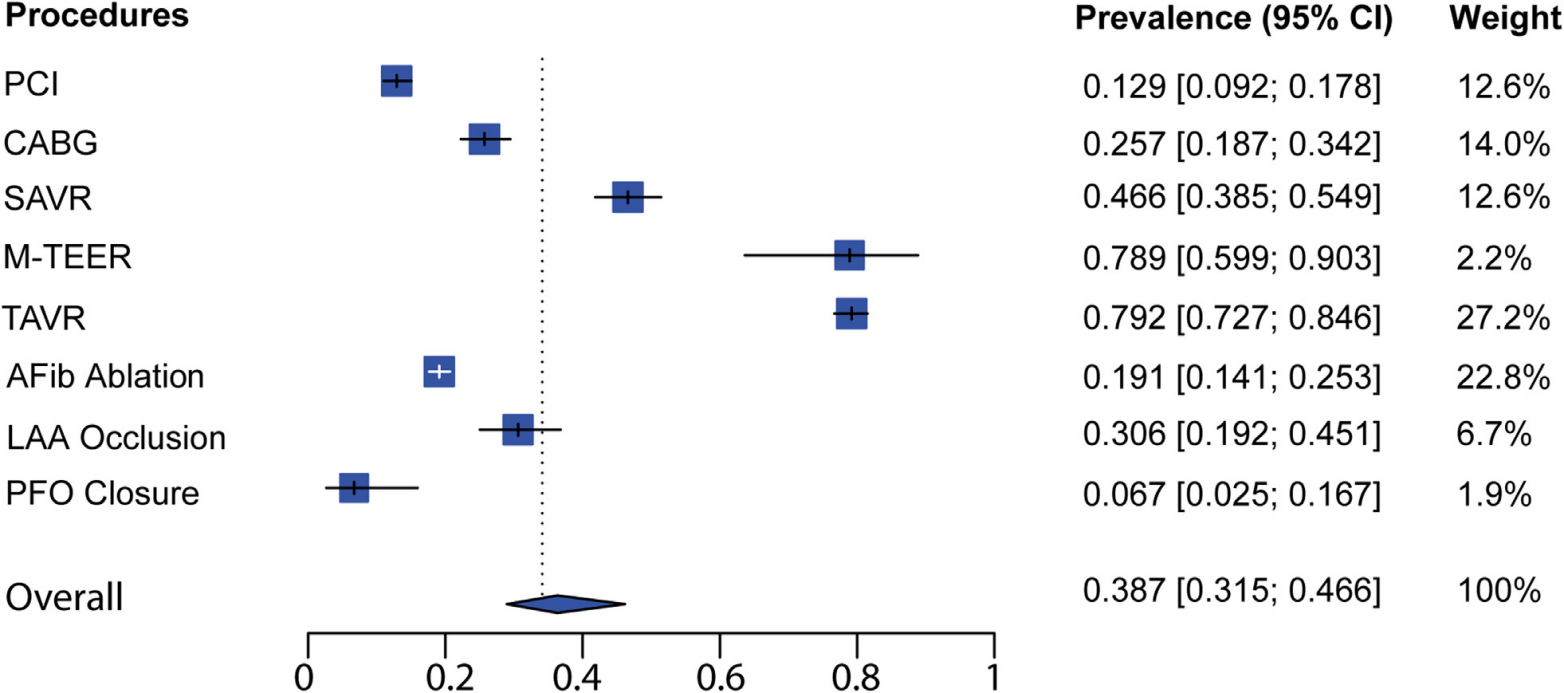
**Prevalence of new ischemic lesions detected by DW-MRI postprocedure.** Pooled estimates by type of procedure and in total (detailed in Supplemental Figure S1). AFib, atrial fibrillation; CABG, coronary artery bypass grafting; DW-MRI, diffusion-weighted magnetic resonance imaging; LAA, left atrial appendage; PFO, patent foramen ovale; PCI, percutaneous coronary intervention; SAVR, surgical aortic valve replacement; TAVR, transcatheter aortic valve replacement; M-TEER, mitral transcatheter edge-to-edge repair.

The literature search identified 80 studies with 88 intervention groups meeting eligibility criteria, which included 6030 patients (Supplemental Appendix). The occurrence of new ischemic lesions detected by DW-MRI (at 1-7 days postprocedure) differs by the type of intervention (Supplemental Appendix). Pooled estimates of the prevalence of DW-MRI lesions for each intervention vary from a low of 6.7% to a high of 79% (Figure 1 and Supplemental Figure S1). On average, across coronary and structural heart procedures with available data, the pooled estimate of prevalence of new ischemic lesions is 38.7% (95% CI, 31.5-46.6) (Figure 1). Among coronary interventions, percutaneous coronary intervention bears a lower risk at 12.9% (95% CI, 9.2-17.8), whereas the risk is nearly doubled with CABG at 25.7% (95% CI, 18.7-34.2) (*P* < .0001). A higher incidence of DW-MRI lesions was identified after structural heart procedures such as TAVR (79.2%; 95% CI, 72.7-84.6), M-TEER (78.9%; 95% CI, 59.9-90.3), and SAVR (46.6%; 95% CI, 38.5-54.9). However, the increased risk with TAVR versus SAVR was statistically significant (*P* < .0001), suggesting that the relatively higher ischemic risk with surgical compared with transcatheter procedures in coronary interventions is reversed in structural interventions. The incidence of cerebral infarcts post-AFib ablation was estimated at 19.1% (95% CI, 14.1-25.3), varying slightly depending on the catheter type, ablation technology, and anticoagulant regimen. The incidence of new ischemic DW-MRI lesions post-LAA occlusion was estimated at 30.6% (95% CI, 19.2-45.1). Compared with that of other structural heart interventions, the frequency of new cerebral lesions after PFO closure was the lowest (6.7%; 95% CI, 2.5-16.7).

When the occurrence of DW-MRI lesions is extrapolated to the annual volume of procedures for each type of intervention and overall counts, of the 2 million patients undergoing cardiac and structural heart procedures in the United States each year, an estimated 330,225 patients (95% CI, 249,948-430,377) will acquire new ischemic lesions postprocedure (Table 2 and Central Illustration). The number is most likely underestimated owing to the lack of prospective systematic neurologic assessments in most studies. Nevertheless, in daily clinical practice, covert stroke is by far the most frequent incidental finding on brain imaging, and its clinical consequences remain poorly defined.^23^ Approximately 40% of these 330,225 patients will acquire new ischemic lesions after CABG, TAVR, SAVR, and M-TEER. Most (60%) of the patients with new ischemic lesions will experience them after PTCA or cardiac catheterization; although the procedure itself has a low risk, it is the most commonly performed cardiac intervention (Table 2 and Central Illustration).

**Table 2:** The estimated number of patients at risk of acquiring new ischemic lesions each year by type of procedure and in total.

Cardiac procedures	No. of annual US patients	Prevalence estimates DW-MRI lesions (%) (95% CI)	Estimated annual US patients with DW-MRI lesions (n) (95% CI)
PCI	1,496,000	12.9 (9.2-17.8)	192,984 (137,632-266,288)
CABG	169,410	25.7 (18.7-34.2)	43,538 (31,680-57,938)
SAVR	37,835	46.6 (38.5-54.9)	17,631 (14,566-20,771)
M-TEER	10,692	78.9 (59.9-90.3)	8436 (6405-9655)
TAVR	72,991	79.2 (72.7-84.6)	57,809 (53,064-61,750)
AFib ablation	22,000	19.1 (14.1-25.3)	4202 (3102-5566)
LAA occlusion	18,000	30.6 (19.2-45.1)	5508 (3456-8118)
PFO closure	1735	6.7 (2.5-16.7)	116 (43-290)
Total	1,828,663		330,225 (249,948-430,377)

Patients at risk undergoing CABG also includes patients undergoing combined procedures with SMVR or MV repair, risk estimate based on CABG; patients at risk undergoing SAVR also includes patients undergoing combined procedures with CABG or SMVR, risk estimate based on SAVR.

AFib, atrial fibrillation; CABG, coronary artery bypass grafting; LAA, left atrial appendage; M-TEER, mitral transcatheter edge-to-edge repair; PFO, patent foramen ovale; PCI, percutaneous coronary intervention; SAVR, surgical aortic valve replacement; SMVR, surgical mitral valve replacement; TAVR, transcatheter aortic valve replacement.

**Central Illustration:**
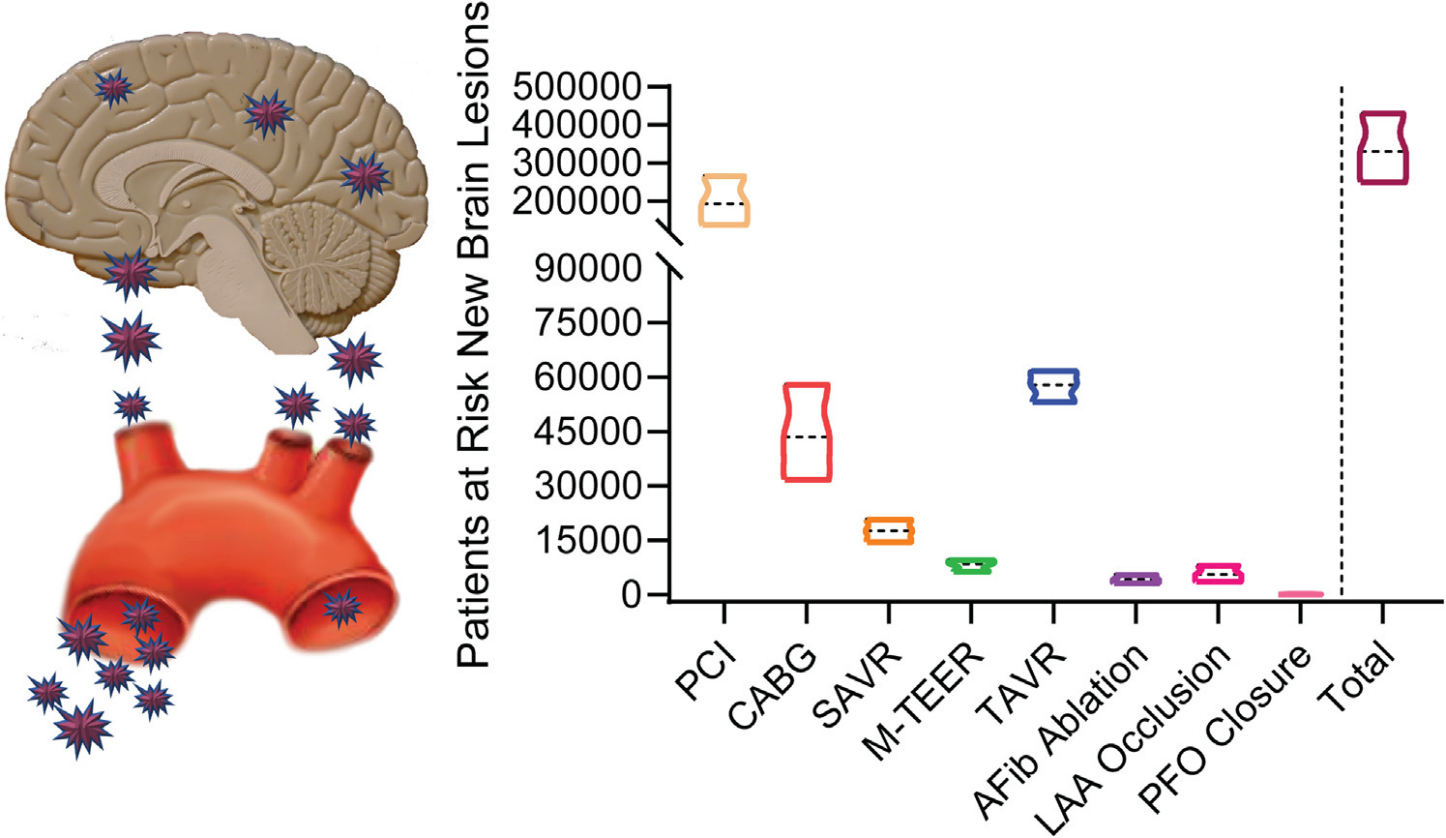
**Ischemic brain injury after coronary and structural heart intervention.** Estimated number of patients at risk of acquiring new ischemic lesions each year by type of intervention and in total. AFib, atrial fibrillation; CABG, coronary artery bypass grafting; LAA, left atrial appendage; PFO, patent foramen ovale; PCI, percutaneous coronary intervention; SAVR, surgical aortic valve replacement; TAVR, transcatheter aortic valve replacement; M-TEER, mitral transcatheter edge-to-edge repair.

## Clinical implications of covert stroke

Ischemic DW-MRI lesions detected postprocedure are more prevalent than symptomatic stroke, and despite the apparent lack of acute symptoms, concerns remain because spontaneous DW-MRI lesions in the population (outside of procedures) have been associated over time with deterioration in cognitive function, increased risk of stroke, dementia, and death.^24^

Despite that up to 50% of patients undergoing surgical procedures experience postoperative cognitive dysfunction,^25^ the clinical impact of ischemic brain injury on neurologic or cognitive function has not been completely characterized. Among studies reporting postoperative DW-MRI lesions (Supplemental Figure S1), 2 studies demonstrated a significant association of ischemic lesions with cognitive decline in psychomotor speed, mental flexibility, memory, and attention at 6 weeks and 3 months postprocedure,^26^^,^^27^ whereas 5 other studies did not demonstrate a significant correlation between the presence of new ischemic lesions and neurocognitive dysfunction early (at discharge or 3 months) or later (at 3 years postprocedure).^28^, ^29^, ^30^, ^31^, ^32^ In a comparative study of CABG and coronary catheterization, the presence of postprocedural ischemic DW-MRI lesions was correlated with a reduced performance in verbal and visual memory at 3 months postprocedure.^33^

A small-scale registry suggested that 1 in every 5 patients will have measurable neurologic impairment after TAVR and 1 in 3 patients will have a decrease in cognitive function using the Montreal Cognitive Assessment associated with the presence of DW-MRI ischemic lesions.^34^ Furthermore, the neurocognitive decline seems to be correlated with the lesion number and volume.^35^, ^36^, ^37^, ^38^ Consistent with this finding, a meta-analysis demonstrated that a higher number of DW-MRI lesions was associated with a greater incidence of early cognitive decline (mean 6.1 ± 1.7 months).^39^ Association of new ischemic lesions with cognitive decline was also evident in intermediate-risk patients, underlining the risk of clinical manifestation of neurologic deficits over time in lower-risk patients.^40^ Several small studies showed a potential effect of ischemic lesions on cognitive decline after M-TEER,^41^ AFib catheter ablation,^42^ and PFO closure.^43^

The evidence of a link between iatrogenic covert stroke and neurocognitive deficits in coronary and structural heart interventions is generally conflicting and has not been systematically evaluated. Comparisons across studies are hampered by inconsistent definitions, methodology, and timing in addition to multiple confounders, such as anesthetic agents and delirium, which acutely affect cognitive status.

Nevertheless, as more younger patients undergo transcatheter procedures with a higher risk of postprocedural DW-MRI lesions, such as TAVR, M-TEER, LAA occlusion, and AFib ablation, the prevention of iatrogenic covert stroke is even more important. Longer life expectancy in these younger patients means longer time to deal with possible neurologic complications such as cognitive decline, dementia, and stroke.

## Cerebral embolic protection

Recently, cerebral embolic protection (CEP) devices have been developed to reduce the risk of embolic debris reaching the brain by positioning a mesh across the cerebral blood vessels to either capture or deflect the material destined for the brain circulation. Currently, the Sentinel CEP (Boston Scientific) is the only FDA-approved device for use during TAVR, but it is currently used in fewer than 13% of procedures.^44^ Although this device is effective in capturing embolic debris, it has not demonstrated a significant reduction in lesion volume on DW-MRI.^35^ A recently published TVT registry propensity-weighted analysis showed a small reduction (18% relative lower) of in-hospital stroke in patients receiving the Sentinel device during TAVR compared with those without the device.^44^ Similarly, a propensity-weighted analysis of the NIS registry showed that Sentinel use was associated with a lower risk of in-hospital ischemic stroke (1% vs 3.8%; OR, 0.24; 95% CI, 0.09-0.62).^45^ Most recently, the PROTECTED TAVR trial, a large-scale randomized trial of Sentinel during TAVR (n = 1501) compared with control without Sentinel protection (n = 1499), showed no significant difference in the occurrence of in-hospital stroke between the groups (2.3% vs 2.9%; *P* = .30), although there was a reduction in the secondary end point of disabling stroke (0.5% vs 1.3%; *P* = .02).^46^ The British Heart Foundation (BHF) PROTECT-TAVI trial, another large clinical outcome trial testing the effect of the Sentinel CEP on symptomatic stroke during TAVR, is now underway (ISRCTN16665769). Several next-generation CEP devices are also currently under evaluation.^47^

## Limitations

This review has some limitations. The incidence of clinical stroke is likely underestimated because many of the registries use voluntary reporting of complications, with a passive retrospective chart review. When stroke rates were not provided in the source for annual number of procedures, a different reference was used instead. The prevalence of covert stroke by DW-MRI was determined by a literature search; although all efforts were made to be comprehensive, the search was not a systematic review of the literature. The pooling estimates might reflect evidence of heterogeneity among the studies regarding the advancement in technology, sensitivity of detection, patient characteristics, and interventional risk. The assumptions and extrapolations used to estimate the numbers of patients at risk might be a source of bias. Antithrombotic coagulation strategies to reduce the risk of stroke were not discussed. Atrial fibrillation is not typically categorized as structural heart disease, but it was included because it is related to structural cardiac alterations.

## Conclusions

Embolic cerebral injury is a complication associated with all cardiac procedures. Symptomatic stroke rates vary by procedure, although the overall rates are generally low. While the prevalence of acute asymptomatic ischemic brain lesions also varies depending on the procedure, they are much more common, although the clinical implications remain speculative at this time. Whether there is an increased risk of subsequent stroke, dementia, and death in the long term after procedural embolic injury, as has been shown with spontaneous ischemic brain lesions, remains unknown. With the increasing use of surgical and endovascular cardiac procedures in clinical practice, the implications of iatrogenic cerebral embolic injury will require a careful consideration of neurocognitive assessments and longer-term follow-up.
