# Supplementary material for: Cerebral Embolic Risk in Coronary and Structural Heart Interventions: Clinical Evidence
Source: J Soc Cardiovasc Angiogr Interv. 2023 Mar 29;2(3):100631. doi: 10.1016/j.jscai.2023.100631 (PMC11307836; doi:10.1016/j.jscai.2023.100631)
Supplement: Supplemental Appendix [file mmc1.docx]

**SUPPLEMENTAL MATERIAL**

**Supplemental Appendix**

**Procedural Covert Stroke (Detected by DW-MRI)**

**Methods**

To estimate the occurrence of covert stroke during cardiac procedures, a literature search was conducted to identify studies reporting new ischemic lesions detected by diffusion-weighted magnetic resonance imaging (DW-MRI) at 1-7 days post-procedure for each type of intervention.

Literature Search Strategy

A literature search was conducted in PubMed and the Cochrane Database of Systematic Reviews to identify studies reporting new ischemic lesions detected by DW-MRI. Studies were included if DW-MRI was used to identify new ischemic lesions in the early postoperative period (1-7 days) following percutaneous coronary interventions (PCI) or coronary angiography, coronary artery bypass grafting (CABG), surgical aortic valve replacement (SAVR), surgical mitral valve replacement (SMVR), mitral valve (MV) repair, transcatheter mitral valve replacement (TMVR), mitral transcatheter edge-to-edge repair (M-TEER), transcatheter aortic valve replacement (TAVR), atrial fibrillation (AFib) ablation, left atrial appendage (LAA) closure, patent foramen ovale (PFO) closure, and atrial septal defect (ASD) closure.

Statistical Analysis

Data on the occurrence of DW-MRI lesions were pooled for each type of intervention and total and pooled estimates with 95% CI were calculated using the inverse-variance random effects model.

**Results**

The literature search resulted in a total of 80 studies with 88 intervention groups meeting eligibility criteria. Among these 88 intervention groups, there were: 11 PCI (n=1046), 12 CABG (n=566), 11 SAVR (n=413), 2 M-TEER (n=40), 25 TAVR (n=1081), 19 AFib ablation (n=2585), 6 LAA closure (n=234), and 2 PFO closure (n=65) for a total of 6030 patients. There were no studies reporting the occurrence of DW-MRI detected lesions following SMVR, MV repair, TMVR, or ASD closure.


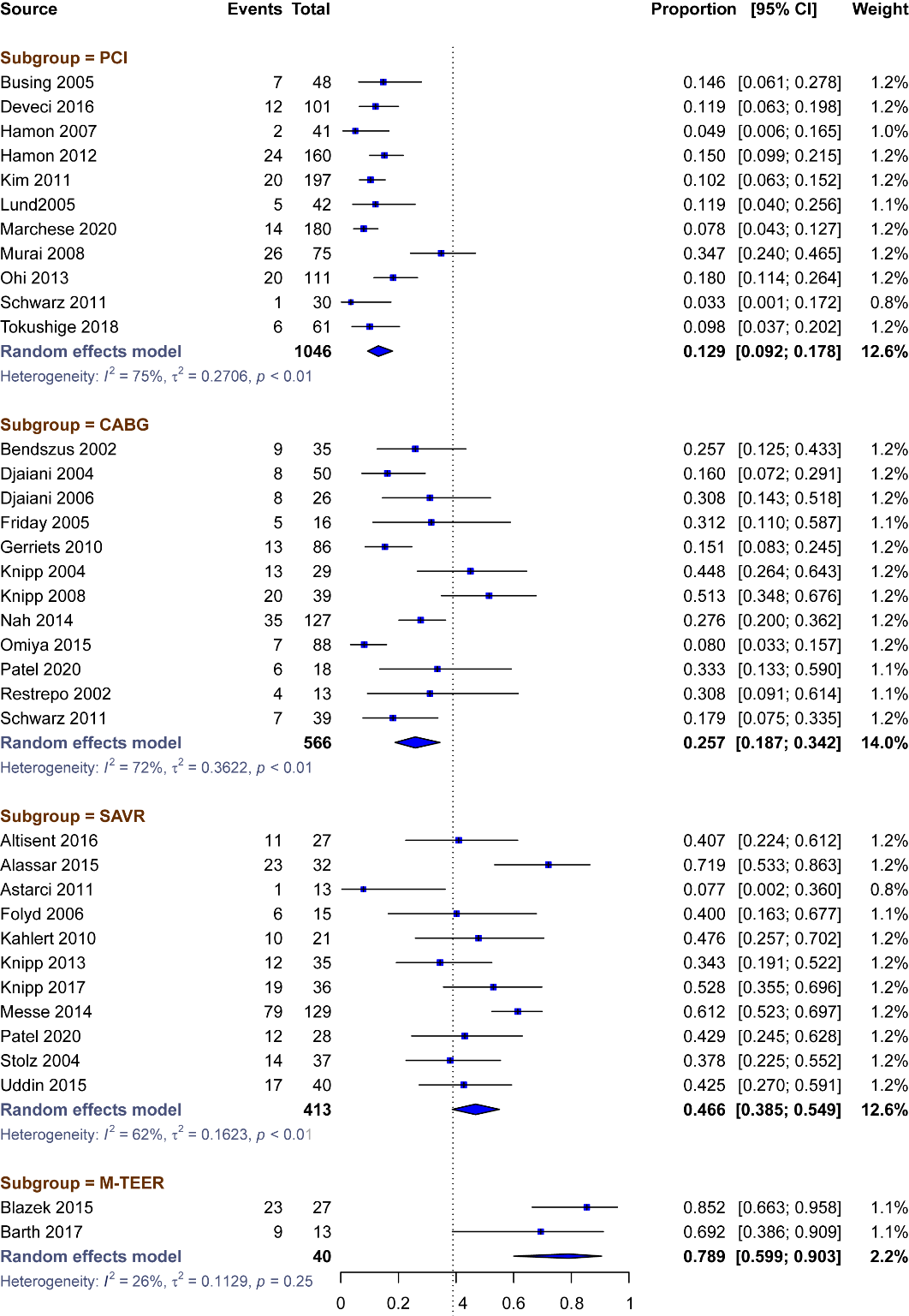
**Supplemental Figure 1.** **Pooled averages of prevalence of new ischemic lesions detected by DW-MRI post procedure per each intervention and total. Inverse-variance random effects model.**


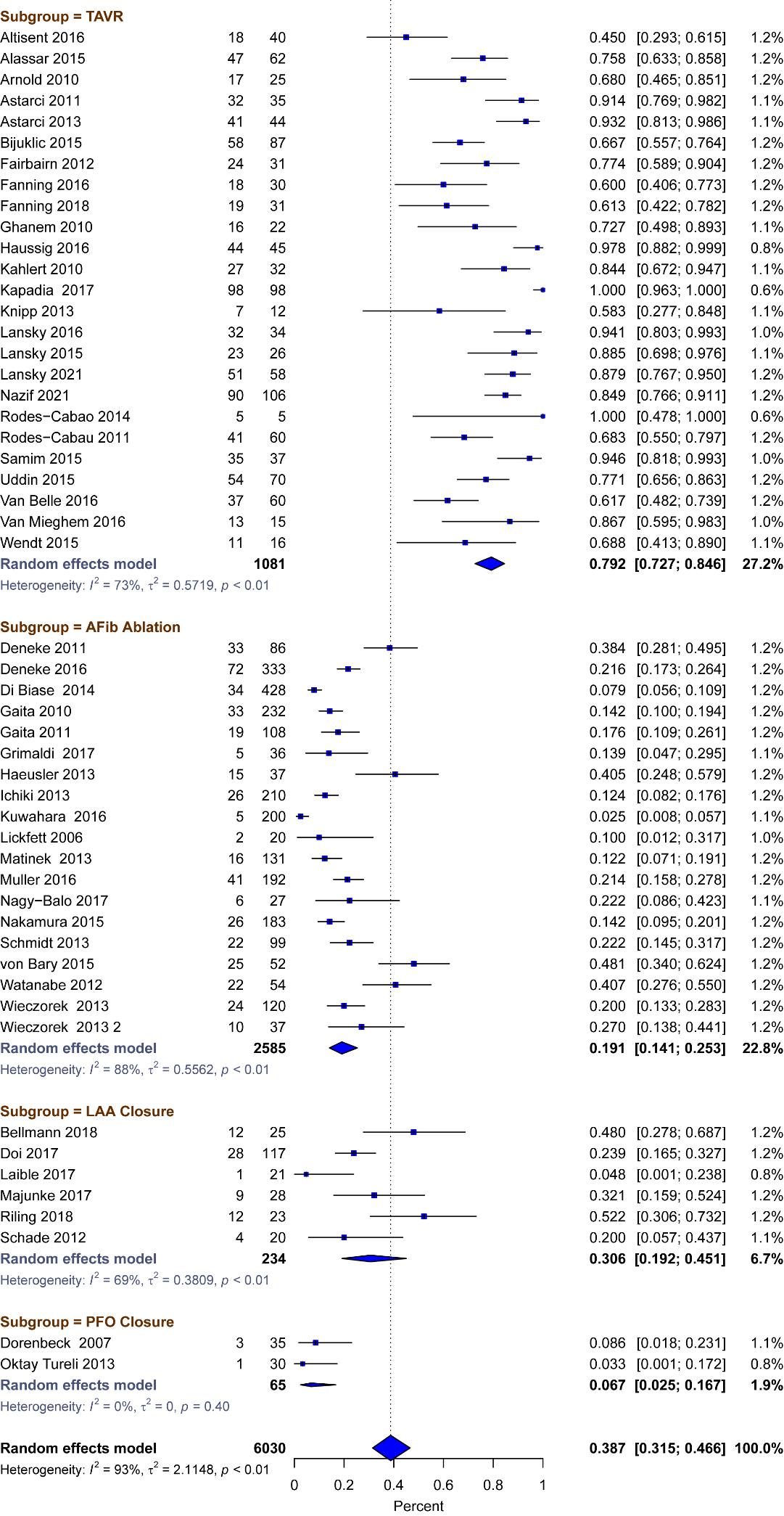


**REFERENCES**

**PCI**

1. Büsing KA, Schulte-Sasse C, Flüchter S, Süselbeck T, Haase KK, Neff W, Hirsch JG, Borggrefe M, Düber C. Cerebral infarction: incidence and risk factors after diagnostic and interventional cardiac catheterization--prospective evaluation at diffusion-weighted MR imaging. *Radiology*. 2005;235:177-83.

2. Deveci OS, Celik AI, Ikikardes F, Ozmen C, Caglıyan CE, Deniz A, Bicakci K, Bicakci S, Evlice A, Demir T, et al. The Incidence and the Risk Factors of Silent Embolic Cerebral Infarction After Coronary Angiography and Percutaneous Coronary Interventions. *Angiology*. 2016;67:433-7.

3. Hamon M, Gomes S, Clergeau MR, Fradin S, Morello R, Hamon M. Risk of acute brain injury related to cerebral microembolism during cardiac catheterization performed by right upper limb arterial access. *Stroke*. 2007;38:2176-9.

4. Hamon M, Lipiecki J, Carrié D, Burzotta F, Durel N, Coutance G, Boudou N, Colosimo C, Trani C, Dumonteil N, et al. Silent cerebral infarcts after cardiac catheterization: a randomized comparison of radial and femoral approaches. *Am Heart J*. 2012;164:449-54.e1.

5. Kim IC, Hur SH, Park NH, Jun DH, Cho YK, Nam CW, Kim H, Han SW, Choi SY, Kim YN, et al. Incidence and predictors of silent embolic cerebral infarction following diagnostic coronary angiography. *Int J Cardiol*. 2011;148:179-82.

6. Lund C, Nes RB, Ugelstad TP, Due-Tonnessen P, Andersen R, Hol PK, Brucher R, Russell D. Cerebral emboli during left heart catheterization may cause acute brain injury. *Eur Heart J*. 2005;26:1269-75.

7. Marchese N, Copetti M, Inchingolo V, Popolizio T, Fontana A, Simeone A, Vigna C. Cerebral Infarcts After Coronary Angiography and Percutaneous Coronary Intervention: A Prospective Propensity-Score-Adjusted Comparison of Right Radial, Left Radial, and Femoral Approaches. *Cardiovasc Revasc Med*. 2020;21:882-87.

8. Murai M, Hazui H, Sugie A, Hoshiga M, Negoro N, Muraoka H, Miyamoto H, Kobata H, Fukumoto H, Ishihara T, et al. Asymptomatic acute ischemic stroke after primary percutaneous coronary intervention in patients with acute coronary syndrome might be caused mainly by manipulating catheters or devices in the ascending aorta, regardless of the approach to the coronary artery. *Circ J*. 2008;72:51-5.

9. Ohi Y, Uno Y, Oohira T, Itakura K, Nishigaki K, Minatoguchi S. Cerebral microembolism following coronary angiography--a prospective comparative study between left cardiac catheterization and multidetector computed tomography. *Intern Med*. 2013;52:1869-74.

10. Schwarz N, Schoenburg M, Mollmann H, Kastaun S, Kaps M, Bachmann G, Sammer G, Hamm C, Walther T, Gerriets T. Cognitive decline and ischemic microlesions after coronary catheterization. A comparison to coronary artery bypass grafting. *Am Heart J*. 2011;162:756-63.

11. Tokushige A, Miyata M, Sonoda T, Kosedo I, Kanda D, Takumi T, Kumagae Y, Fukukura Y, Ohishi M. Prospective Study on the Incidence of Cerebrovascular Disease After Coronary Angiography. *J Atheroscler Thromb*. 2018;25:224-32.

**CABG**

1. Bendszus M, Reents W, Franke D, Mullges W, Babin-Ebell J, Koltzenburg M, Warmuth-Metz M, Solymosi L. Brain damage after coronary artery bypass grafting. *Arch Neurol*. 2002;59:1090-5.

2. Djaiani G, Fedorko L, Borger M, Mikulis D, Carroll J, Cheng D, Karkouti K, Beattie S, Karski J. Mild to moderate atheromatous disease of the thoracic aorta and new ischemic brain lesions after conventional coronary artery bypass graft surgery. *Stroke*. 2004;35:e356-8.

3. Djaiani G, Fedorko L, Cusimano RJ, Mikulis D, Carroll J, Poonawala H, Beattie S, Karski J. Off-pump coronary bypass surgery: risk of ischemic brain lesions in patients with atheromatous thoracic aorta. *Can J Anaesth*. 2006;53:795-801.

4. Friday G, Sutter F, Curtin A, Kenton E, Caplan B, Nocera R, Siddiqui A, Goldman S. Brain magnetic resonance imaging abnormalities following off-pump cardiac surgery. *Heart Surg Forum*. 2005;8:E105-9.

5. Gerriets T, Schwarz N, Bachmann G, Kaps M, Kloevekorn WP, Sammer G, Tschernatsch M, Nottbohm R, Blaes F, Schonburg M. Evaluation of methods to predict early long-term neurobehavioral outcome after coronary artery bypass grafting. *Am J Cardiol*. 2010;105:1095-101.

6. Knipp SC, Matatko N, Wilhelm H, Schlamann M, Massoudy P, Forsting M, Diener HC, Jakob H. Evaluation of brain injury after coronary artery bypass grafting. A prospective study using neuropsychological assessment and diffusion-weighted magnetic resonance imaging. *Eur J Cardiothorac Surg*. 2004;25:791-800.

7. Knipp SC, Matatko N, Wilhelm H, Schlamann M, Thielmann M, Losch C, Diener HC, Jakob H. Cognitive outcomes three years after coronary artery bypass surgery: relation to diffusion-weighted magnetic resonance imaging. *Ann Thorac Surg*. 2008;85:872-9.

8. Nah HW, Lee JW, Chung CH, Choo SJ, Kwon SU, Kim JS, Warach S, Kang DW. New brain infarcts on magnetic resonance imaging after coronary artery bypass graft surgery: lesion patterns, mechanism, and predictors. *Ann Neurol*. 2014;76:347-55.

9. Omiya H, Yoshitani K, Yamada N, Kubota Y, Takahashi K, Kobayashi J, Ohnishi Y. Preoperative brain magnetic resonance imaging and postoperative delirium after off-pump coronary artery bypass grafting: a prospective cohort study. *Can J Anaesth*. 2015;62:595-602.

10. Patel N, Banahan C, Janus J, Horsfield MA, Cox A, Marshall D, Colman J, Morlese J, Evans DH, Hannon C, et al. Neurological impact of emboli during adult cardiac surgery. *J Neurol Sci*. 2020;416:117006.

11. Restrepo L, Wityk RJ, Grega MA, Borowicz L, Jr., Barker PB, Jacobs MA, Beauchamp NJ, Hillis AE, McKhann GM. Diffusion- and perfusion-weighted magnetic resonance imaging of the brain before and after coronary artery bypass grafting surgery. *Stroke*. 2002;33:2909-15.

12. Schwarz N, Schoenburg M, Mollmann H, Kastaun S, Kaps M, Bachmann G, Sammer G, Hamm C, Walther T, Gerriets T. Cognitive decline and ischemic microlesions after coronary catheterization. A comparison to coronary artery bypass grafting. *Am Heart J*. 2011;162:756-63.

**SAVR**

1. Abdul-Jawad Altisent O, Ferreira-Gonzalez I, Marsal JR, Ribera A, Auger C, Ortega G, Cascant P, Urena M, Del Blanco BG, Serra V, et al. Neurological damage after transcatheter aortic valve implantation compared with surgical aortic valve replacement in intermediate risk patients. *Clin Res Cardiol*. 2016;105:508-17.

2. Alassar A, Soppa G, Edsell M, Rich P, Roy D, Chis Ster I, Joyce R, Valencia O, Barrick T, Howe F, et al. Incidence and mechanisms of cerebral ischemia after transcatheter aortic valve implantation compared with surgical aortic valve replacement. *Ann Thorac Surg*. 2015;99:802-8.

3. Astarci P, Glineur D, Kefer J, D'Hoore W, Renkin J, Vanoverschelde JL, El Khoury G, Grandin C. Magnetic resonance imaging evaluation of cerebral embolization during percutaneous aortic valve implantation: comparison of transfemoral and trans-apical approaches using Edwards Sapiens valve. *European Journal of Cardio-Thoracic Surgery*. 2011;40:475-79.

4. Floyd TF, Shah PN, Price CC, Harris F, Ratcliffe SJ, Acker MA, Bavaria JE, Rahmouni H, Kuersten B, Wiegers S, et al. Clinically silent cerebral ischemic events after cardiac surgery: their incidence, regional vascular occurrence, and procedural dependence. *Ann Thorac Surg*. 2006;81:2160-6.

5. Kahlert P, Knipp SC, Schlamann M, Thielmann M, Al-Rashid F, Weber M, Johansson U, Wendt D, Jakob HG, Forsting M, et al. Silent and apparent cerebral ischemia after percutaneous transfemoral aortic valve implantation: a diffusion-weighted magnetic resonance imaging study. *Circulation*. 2010;121:870-8.

6. Knipp SC, Kahlert P, Jokisch D, Schlamann M, Wendt D, Weimar C, Jakob H, Thielmann M. Cognitive function after transapical aortic valve implantation: a single-centre study with 3-month follow-up. *Interact Cardiovasc Thorac Surg*. 2013;16:116-22.

7. Knipp SC, Weimar C, Schlamann M, Schweter S, Wendt D, Thielmann M, Benedik J, Jakob H. Early and long-term cognitive outcome after conventional cardiac valve surgery. *Interact Cardiovasc Thorac Surg*. 2017;24:534-40.

8. Messé SR, Acker MA, Kasner SE, Fanning M, Giovannetti T, Ratcliffe SJ, Bilello M, Szeto WY, Bavaria JE, Hargrove WC, 3rd, et al. Stroke after aortic valve surgery: results from a prospective cohort. *Circulation*. 2014;129:2253-61.

9. Patel N, Banahan C, Janus J, Horsfield MA, Cox A, Marshall D, Colman J, Morlese J, Evans DH, Hannon C, et al. Neurological impact of emboli during adult cardiac surgery. *J Neurol Sci*. 2020;416:117006.

10. Stolz E, Gerriets T, Kluge A, Klovekorn WP, Kaps M, Bachmann G. Diffusion-weighted magnetic resonance imaging and neurobiochemical markers after aortic valve replacement: implications for future neuroprotective trials? *Stroke*. 2004;35:888-92.

11. Uddin A, Fairbairn TA, Djoukhader IK, Igra M, Kidambi A, Motwani M, Herzog B, Ripley DP, Musa TA, Goddard AJ, et al. Consequence of cerebral embolism after transcatheter aortic valve implantation compared with contemporary surgical aortic valve replacement: effect on health-related quality of life. *Circ Cardiovasc Interv*. 2015;8:e001913.

**M-TEER**

1. Barth S, Hamm K, Fodor S, Reents W, Kerber S, Halbfass P, Hautmann MB, Schieffer B, Soda H. Incidence and Clinical Impact of Cerebral Lesions after the MitraClipA(R) Procedure. *J Heart Valve Dis*. 2017;26:175-84.

2. Blazek S, Lurz P, Mangner N, Fuernau G, Seeburger J, Luecke C, Gutberlet M, Ender J, Desch S, Eitel I, et al. Incidence, characteristics and functional implications of cerebral embolic lesions after the MitraClip procedure. *EuroIntervention*. 2015;10:1195-203.

**TAVR**

1. Abdul-Jawad Altisent O, Ferreira-Gonzalez I, Marsal JR, Ribera A, Auger C, Ortega G, Cascant P, Urena M, Del Blanco BG, Serra V, et al. Neurological damage after transcatheter aortic valve implantation compared with surgical aortic valve replacement in intermediate risk patients. *Clin Res Cardiol*. 2016;105:508-17.

2. Alassar A, Soppa G, Edsell M, Rich P, Roy D, Chis Ster I, Joyce R, Valencia O, Barrick T, Howe F, et al. Incidence and mechanisms of cerebral ischemia after transcatheter aortic valve implantation compared with surgical aortic valve replacement. *Ann Thorac Surg*. 2015;99:802-8.

3. Arnold M, Schulz-Heise S, Achenbach S, Ott S, Dorfler A, Ropers D, Feyrer R, Einhaus F, Loders S, Mahmoud F, et al. Embolic cerebral insults after transapical aortic valve implantation detected by magnetic resonance imaging. *JACC Cardiovasc Interv*. 2010;3:1126-32.

4. Astarci P, Glineur D, Kefer J, D'Hoore W, Renkin J, Vanoverschelde JL, El Khoury G, Grandin C. Magnetic resonance imaging evaluation of cerebral embolization during percutaneous aortic valve implantation: comparison of transfemoral and trans-apical approaches using Edwards Sapiens valve. *European Journal of Cardio-Thoracic Surgery*. 2011;40:475-79.

5. Astarci P, Price J, Glineur D, D'Hoore W, Kefer J, Elkhoury G, Grandin C, Vanoverschelde JL. Cerebral embolization during percutaneous valve implantation does not occur during balloon inflation valvuloplasty: prospective diffusion-weighted brain MRI study. *J Heart Valve Dis*. 2013;22:79-84.

6. Bijuklic K, Haselbach T, Witt J, Krause K, Hansen L, Gehrckens R, Riess FC, Schofer J. Increased Risk of Cerebral Embolization After Implantation of a Balloon-Expandable Aortic Valve Without Prior Balloon Valvuloplasty. *JACC Cardiovasc Interv*. 2015;8:1608-13.

7. Fairbairn TA, Mather AN, Bijsterveld P, Worthy G, Currie S, Goddard AJ, Blackman DJ, Plein S, Greenwood JP. Diffusion-weighted MRI determined cerebral embolic infarction following transcatheter aortic valve implantation: assessment of predictive risk factors and the relationship to subsequent health status. *Heart*. 2012;98:18-23.

8. Fanning JP, Wesley AJ, Walters DL, Eeles EM, Barnett AG, Platts DG, Clarke AJ, Wong AA, Strugnell WE, O'Sullivan C, et al. Neurological Injury in Intermediate-Risk Transcatheter Aortic Valve Implantation. *J Am Heart Assoc*. 2016;5.

9. Fanning JP, Wesley AJ, Walters DL, Wong AA, Barnett AG, Strugnell WE, Platts DG, Fraser JF. Topographical distribution of perioperative cerebral infarction associated with transcatheter aortic valve implantation. *Am Heart J*. 2018;197:113-23.

10. Ghanem A, Muller A, Nahle CP, Kocurek J, Werner N, Hammerstingl C, Schild HH, Schwab JO, Mellert F, Fimmers R, et al. Risk and fate of cerebral embolism after transfemoral aortic valve implantation: a prospective pilot study with diffusion-weighted magnetic resonance imaging. *J Am Coll Cardiol*. 2010;55:1427-32.

11. Haussig S, Mangner N, Dwyer MG, Lehmkuhl L, Lucke C, Woitek F, Holzhey DM, Mohr FW, Gutberlet M, Zivadinov R, et al. Effect of a Cerebral Protection Device on Brain Lesions Following Transcatheter Aortic Valve Implantation in Patients With Severe Aortic Stenosis: The CLEAN-TAVI Randomized Clinical Trial. *JAMA*. 2016;316:592-601.

12. Kahlert P, Knipp SC, Schlamann M, Thielmann M, Al-Rashid F, Weber M, Johansson U, Wendt D, Jakob HG, Forsting M, et al. Silent and apparent cerebral ischemia after percutaneous transfemoral aortic valve implantation: a diffusion-weighted magnetic resonance imaging study. *Circulation*. 2010;121:870-8.

13. Kapadia SR, Kodali S, Makkar R, Mehran R, Lazar RM, Zivadinov R, Dwyer MG, Jilaihawi H, Virmani R, Anwaruddin S, et al. Protection Against Cerebral Embolism During Transcatheter Aortic Valve Replacement. *J Am Coll Cardiol*. 2017;69:367-77.

14. Knipp SC, Kahlert P, Jokisch D, Schlamann M, Wendt D, Weimar C, Jakob H, Thielmann M. Cognitive function after transapical aortic valve implantation: a single-centre study with 3-month follow-up. *Interact Cardiovasc Thorac Surg*. 2013;16:116-22.

15. Lansky AJ, Brown D, Pena C, Pietras CG, Parise H, Ng VG, Meller S, Abrams KJ, Cleman M, Margolis P, et al. Neurologic Complications of Unprotected Transcatheter Aortic Valve Implantation (from the Neuro-TAVI Trial). *Am J Cardiol*. 2016;118:1519-26.

16. Lansky AJ, Makkar R, Nazif T, Messe S, Forrest J, Sharma R, Schofer J, Linke A, Brown D, Dhoble A, et al. A randomized evaluation of the TriGuard HDH cerebral embolic protection device to Reduce the Impact of Cerebral Embolic LEsions after TransCatheter Aortic Valve ImplanTation: the REFLECT I trial. *Eur Heart J*. 2021;42:2670-79.

17. Lansky AJ, Schofer J, Tchetche D, Stella P, Pietras CG, Parise H, Abrams K, Forrest JK, Cleman M, Reinohl J, et al. A prospective randomized evaluation of the TriGuard HDH embolic DEFLECTion device during transcatheter aortic valve implantation: results from the DEFLECT III trial. *Eur Heart J*. 2015;36:2070-78.

18. Nazif TM, Moses J, Sharma R, Dhoble A, Rovin J, Brown D, Horwitz P, Makkar R, Stoler R, Forrest J, et al. Randomized Evaluation of TriGuard 3 Cerebral Embolic Protection After Transcatheter Aortic Valve Replacement: REFLECT II. *JACC Cardiovasc Interv*. 2021;14:515-27.

19. Rodes-Cabau J, Dumont E, Boone RH, Larose E, Bagur R, Gurvitch R, Bedard F, Doyle D, De Larochelliere R, Jayasuria C, et al. Cerebral embolism following transcatheter aortic valve implantation: comparison of transfemoral and transapical approaches. *J Am Coll Cardiol*. 2011;57:18-28.

20. Rodes-Cabau J, Kahlert P, Neumann FJ, Schymik G, Webb JG, Amarenco P, Brott T, Garami Z, Gerosa G, Lefevre T, et al. Feasibility and exploratory efficacy evaluation of the Embrella Embolic Deflector system for the prevention of cerebral emboli in patients undergoing transcatheter aortic valve replacement: the PROTAVI-C pilot study. *JACC Cardiovasc Interv*. 2014;7:1146-55.

21. Samim M, Agostoni P, Hendrikse J, Budde RP, Nijhoff F, Kluin J, Ramjankhan F, Doevendans PA, Stella PR. Embrella embolic deflection device for cerebral protection during transcatheter aortic valve replacement. *J Thorac Cardiovasc Surg*. 2015;149:799-805 e1-2.

22. Uddin A, Fairbairn TA, Djoukhader IK, Igra M, Kidambi A, Motwani M, Herzog B, Ripley DP, Musa TA, Goddard AJ, et al. Consequence of cerebral embolism after transcatheter aortic valve implantation compared with contemporary surgical aortic valve replacement: effect on health-related quality of life. *Circ Cardiovasc Interv*. 2015;8:e001913.

23. Van Belle E, Hengstenberg C, Lefevre T, Kupatt C, Debry N, Husser O, Pontana F, Kuchcinski G, Deliargyris EN, Mehran R, et al. Cerebral Embolism During Transcatheter Aortic Valve Replacement: The BRAVO-3 MRI Study. *J Am Coll Cardiol*. 2016;68:589-99.

24. Van Mieghem NM, van Gils L, Ahmad H, van Kesteren F, van der Werf HW, Brueren G, Storm M, Lenzen M, Daemen J, van den Heuvel AF, et al. Filter-based cerebral embolic protection with transcatheter aortic valve implantation: the randomised MISTRAL-C trial. *EuroIntervention*. 2016;12:499-507.

25. Wendt D, Kleinbongard P, Knipp S, Al-Rashid F, Gedik N, El Chilali K, Schweter S, Schlamann M, Kahlert P, Neuhauser M, et al. Intraaortic Protection From Embolization in Patients Undergoing Transaortic Transcatheter Aortic Valve Implantation. *Ann Thorac Surg*. 2015;100:686-91.

**AFib Ablation**

1. Deneke T, Nentwich K, Halbfass P, Fochler F, Roos M, Grewe P. Asymptomatic acute complications of atrial fibrillation ablation using irrigated radiofrequency technology. *J Am Coll Cardiol*. 2016;1):760.

2. Deneke T, Shin DI, Balta O, Bunz K, Fassbender F, Mugge A, Anders H, Horlitz M, Pasler M, Karthikapallil S, et al. Postablation asymptomatic cerebral lesions: long-term follow-up using magnetic resonance imaging. *Heart Rhythm*. 2011;8:1705-11.

3. Di Biase L, Gaita F, Toso E, Santangeli P, Mohanty P, Rutledge N, Yan X, Mohanty S, Trivedi C, Bai R, et al. Does periprocedural anticoagulation management of atrial fibrillation affect the prevalence of silent thromboembolic lesion detected by diffusion cerebral magnetic resonance imaging in patients undergoing radiofrequency atrial fibrillation ablation with open irrigated catheters? Results from a prospective multicenter study. *Heart Rhythm*. 2014;11:791-8.

4. Gaita F, Caponi D, Pianelli M, Scaglione M, Toso E, Cesarani F, Boffano C, Gandini G, Valentini MC, De Ponti R, et al. Radiofrequency catheter ablation of atrial fibrillation: a cause of silent thromboembolism? Magnetic resonance imaging assessment of cerebral thromboembolism in patients undergoing ablation of atrial fibrillation. *Circulation*. 2010;122:1667-73.

5. Gaita F, Leclercq JF, Schumacher B, Scaglione M, Toso E, Halimi F, Schade A, Froehner S, Ziegler V, Sergi D, et al. Incidence of silent cerebral thromboembolic lesions after atrial fibrillation ablation may change according to technology used: comparison of irrigated radiofrequency, multipolar nonirrigated catheter and cryoballoon. *J Cardiovasc Electrophysiol*. 2011;22:961-8.

6. Grimaldi M, Swarup V, DeVille B, Sussman J, Jais P, Gaita F, Duytschaever M, Ng GA, Daoud E, Lakkireddy DD, et al. Importance of anticoagulation and postablation silent cerebral lesions: Subanalyses of REVOLUTION and reMARQable studies. *Pacing Clin Electrophysiol*. 2017;40:1432-39.

7. Haeusler KG, Koch L, Herm J, Kopp UA, Heuschmann PU, Endres M, Schultheiss HP, Schirdewan A, Fiebach JB. 3 Tesla MRI-detected brain lesions after pulmonary vein isolation for atrial fibrillation: results of the MACPAF study. *J Cardiovasc Electrophysiol*. 2013;24:14-21.

8. Ichiki H, Oketani N, Ishida S, Iriki Y, Okui H, Maenosono R, Namino F, Ninomiya Y, Miyata M, Hamasaki S, et al. The incidence of asymptomatic cerebral microthromboembolism after atrial fibrillation ablation: comparison of warfarin and dabigatran. *Pacing Clin Electrophysiol*. 2013;36:1328-35.

9. Kuwahara T, Abe M, Yamaki M, Fujieda H, Abe Y, Hashimoto K, Ishiba M, Sakai H, Hishikari K, Takigawa M, et al. Apixaban versus Warfarin for the Prevention of Periprocedural Cerebral Thromboembolism in Atrial Fibrillation Ablation: Multicenter Prospective Randomized Study. *J Cardiovasc Electrophysiol*. 2016;27:549-54.

10. Lickfett L, Hackenbroch M, Lewalter T, Selbach S, Schwab JO, Yang A, Balta O, Schrickel J, Bitzen A, Luderitz B, et al. Cerebral diffusion-weighted magnetic resonance imaging: a tool to monitor the thrombogenicity of left atrial catheter ablation. *J Cardiovasc Electrophysiol*. 2006;17:1-7.

11. Martinek M, Sigmund E, Lemes C, Derndorfer M, Aichinger J, Winter S, Jauker W, Gschwendtner M, Nesser HJ, Purerfellner H. Asymptomatic cerebral lesions during pulmonary vein isolation under uninterrupted oral anticoagulation. *Europace*. 2013;15:325-31.

12. Muller P, Halbfass P, Szollosi A, Dietrich JW, Fochler F, Nentwich K, Roos M, Krug J, Schmitt R, Mugge A, et al. Impact of periprocedural anticoagulation strategy on the incidence of new-onset silent cerebral events after radiofrequency catheter ablation of atrial fibrillation. *J Interv Card Electrophysiol*. 2016;46:203-11.

13. Nagy-Baló E, Martirosyan M, Sándorfi G, Hajas O, Lánczi L, Berényi E, Ladányi L, Kiss A, Édes I, Csanádi Z. Cerebral micro-embolization during pulmonary vein isolation: Relation to post-ablation silent cerebral ischemia. *Cardiol J*. 2017;24:234-41.

14. Nakamura K, Naito S, Minami K, Sasaki T, Goto E, Yamaguchi Y, Shimizu S, Senga M, Yano T, Take Y, et al. Silent Cerebral Ischemic Lesions After Atrial Fibrillation Ablation Among Oral Anticoagulants: Predictors of Diffusion-Weighted Imaging-Positive Lesions and Follow-up Study. *Circulation*. 2015;132.

15. Schmidt B, Gunawardene M, Krieg D, Bordignon S, Furnkranz A, Kulikoglu M, Herrmann W, Chun KR. A prospective randomized single-center study on the risk of asymptomatic cerebral lesions comparing irrigated radiofrequency current ablation with the cryoballoon and the laser balloon. *J Cardiovasc Electrophysiol*. 2013;24:869-74.

16. von Bary C, Deneke T, Arentz T, Schade A, Lehrmann H, Eissnert C, Schwab-Malek S, Fredersdorf S, Ücer E, Baldaranov D, et al. Silent cerebral events as a result of left atrial catheter ablation do not cause neuropsychological sequelae--a MRI-controlled multicenter study. *J Interv Card Electrophysiol*. 2015;43:217-26.

17. Watanabe N, Oonuma Y, Kikuchi M, Itou H, Miyoshi F, Adachi T, Asano T, Tanno K, Kobayashi Y. Comparison of new silent cerebral thromboembolic lesions after atrial fibrillation ablation of Pulmonary Vein Isolation with those of complex fractionated atrial electrograms ablation. *Eur Heart J*. 2012;33:215-15.

18. Wieczorek M, Hoeltgen R, Brueck M. Does the number of simultaneously activated electrodes during phased RF multielectrode ablation of atrial fibrillation influence the incidence of silent cerebral microembolism? *Heart Rhythm*. 2013;10:953-9.

19. Wieczorek M, Lukat M, Hoeltgen R, Condie C, Hilje T, Missler U, Hirsch J, Scharf C. Investigation into causes of abnormal cerebral MRI findings following PVAC duty-cycled, phased RF ablation of atrial fibrillation. *J Cardiovasc Electrophysiol*. 2013;24:121-8.

**LAA Closure**

1. Bellmann B, Rillig A, Skurk C, Leistner DM, Haeusler KG, Lin T, Geran R, Koehler L, Guttmann S, Tscholl V, et al. Long-term follow up of 3 T MRI-detected brain lesions after percutaneous catheter-based left atrial appendage closure. *Catheter Cardiovasc Interv*. 2018;92:327-33.

2. Doi A, Takagi M, Kakihara J, Hayashi Y, Tatsumi H, Fujimoto K, Sugioka K, Yoshiyama M. Incidence and predictors of silent cerebral thromboembolic lesions after catheter ablation for atrial fibrillation in patients treated with direct oral anticoagulants. *Heart Vessels*. 2017;32:1227-35.

3. Laible M, Mohlenbruch M, Horstmann S, Pfaff J, Geis NA, Pleger S, Schuler S, Rizos T, Bendszus M, Veltkamp R. Peri-procedural silent cerebral infarcts after left atrial appendage occlusion. *Eur J Neurol*. 2017;24:53-57.

4. Majunke N, Eplinius F, Gutberlet M, Moebius-Winkler S, Daehnert I, Grothoff M, Schurer S, Mangner N, Lurz P, Erbs S, et al. Frequency and clinical course of cerebral embolism in patients undergoing transcatheter left atrial appendage closure. *EuroIntervention*. 2017;13:124-30.

5. Rillig A, Bellmann B, Skurk C, Leistner DM, Haeusler KG, Lin T, Geran R, Koehler L, Guttmann S, Steffens D, et al. Left atrial appendage angiography is associated with the incidence and number of magnetic resonance imaging-detected brain lesions after percutaneous catheter-based left atrial appendage closure. *Heart Rhythm*. 2018;15:3-8.

6. Schade A, Krug J, Stahl C, Szollosi GA, Fodor S, Schmitt R, Ziegler V, Soda H, Deneke T. Left atrial appendage occlusion using the watchman device in a high risk population: Is there a significant risk of periprocedural cerebral stroke or microembolism? *Heart Rhythm*. 2012;1):S317.

**PFO Closure**

1. Dorenbeck U, Simon B, Skowasch D, Stusser C, Gockel A, Schild HH, Urbach H, Bauriedel G. Cerebral embolism with interventional closure of symptomatic patent foramen ovale: an MRI-based study using diffusion-weighted imaging. *Eur J Neurol*. 2007;14:451-4.

2. Oktay Tureli H, Ungan I, Tureli D, Demir B, Pirhan O, Bayrak HI, Caglar IM, Karakaya O, Inci E. Risk of cerebral embolism after interventional closure of symptomatic patent foramen ovale or atrial septal defect: a diffusion-weighted MRI and neuron-specific enolase-based study. *J Invasive Cardiol*. 2013;25:519-24.
